# Cerebrospinal Fluid Cytokines in Patients with Neurosyphilis: The Significance of Interleukin-10 for the Disease

**DOI:** 10.1155/2020/3812671

**Published:** 2020-10-01

**Authors:** Wurong Li, Wenqing Wu, Haoxiao Chang, Meijuan Jiang, Junhua Gao, Yun Xu, Dongmei Xu, Linlin Yin, Xinghu Zhang

**Affiliations:** ^1^Department of Neurology, Beijing Tiantan Hospital, Capital Medical University, Beijing, China; ^2^Advanced Innovation Center for Human Brain Protection, China National Clinical Research Center for Neurological Diseases, Beijing, China; ^3^Department of Neurology, Beijing Ditan Hospital, Capital Medical University, Beijing, China

## Abstract

The aim of this study was to examine the cerebrospinal fluid (CSF) concentrations of proinflammatory and anti-inflammatory cytokines in neurosyphilis (NS), analyze the differences between asymptomatic NS (ANS) and symptomatic NS (SNS), and explore the diagnostic value of these cytokines. We enrolled 45 patients with a diagnosis of NS, including 18 patients with ANS and 27 patients with SNS, whose cerebrospinal fluid (CSF) samples were collected before penicillin therapy. Twelve patients with syphilis but non-NS (NNS) were also included. We measured the CSF levels of interleukin- (IL-) 1*β*, IL-4, IL-6, IL-10, IL-17A, IL-21, and tumor necrosis factor- (TNF-) *α*; the CSF levels of the microglial activation marker soluble triggering receptor expressed on myeloid cells 2 (sTREM2); and the CSF levels of the neuronal injury marker neurofilament light proteins (NFL) using the human cytokine multiplex assay or ELISA. Of the measured cytokines in the CSF, only IL-10 levels were significantly increased in NS patients compared to NNS patients (*p* < 0.001). In a subgroup analysis, the CSF levels of IL-10 were significantly elevated in SNS patients compared to ANS and NNS patients (*p* = 0.024 and *p* < 0.001, respectively). The CSF IL-10 levels had a significant correlation with the markers of microglial activation and neuronal injury, and they also correlated with CSF rapid plasma reagin (RPR) titer, CSF white blood cell (WBC) count, and CSF protein concentration. The areas under the ROC curve (AUC) of CSF IL-10 in the diagnosis of NS and ANS were 0.920 and 0.891, respectively. The corresponding sensitivities/specificities were 86.7%/91.7% and 83.3%/91.7%, respectively. Therefore, the excessive production of IL-10 might facilitate bacterial persistent infection, play an important role in the pathogenesis of NS, and associate with the progression of the disease. CSF IL-10 concentration had a useful value in the diagnosis of NS, especially in ANS.

## 1. Introduction

Syphilis is a chronic and systemic sexually transmitted disease caused by *Treponema pallidum* infection, which can induce chronic inflammation to the cardiovascular system, the skeletal system, the nervous system, and other human organ systems [1, 2]. Neurosyphilis can occur at any time in the course of the infection, and it may affect the meninges, brain, and spinal cord and may be asymptomatic or symptomatic [3, 4]. CSF analysis of syphilis patients showed that *T. pallidum* could invade the CSF early in the course of disease [5]. It was believed that *T. pallidum* could be removed from the CSF by itself in most patients, while those who failed to remove *T. pallidum* were asymptomatic neurosyphilis patients (ANS) and may developed into SNS [6, 7]. However, the pathogenesis of NS is largely unknown, and the mechanisms underlying the disease progression in some patients (i.e., to SNS) are poorly understood.

The cell-mediated immune response plays an important role in the natural course of *T. pallidum* infection. *T. pallidum* infection can cause a strong host-specific immune response as well as the secretion of a large number of proinflammatory cytokines to clear the spirochetes [8, 9]. However, the host's excess inflammatory and immune response contributes to syphilis-related symptoms and tissue damage [10]. Thus, the anti-inflammatory immune response here seems to be important in balancing the excessive proinflammatory response.

In this report, we detected proinflammatory (IL-1*β*, IL-6, IL-17A, IL-21, and TNF-*α*) and anti-inflammatory (IL-4 and IL-10) cytokines in patients with SNS, ANS, and NNS and determined their association with markers of microglia activation and neuronal injury (sTREM2 and NFL), aiming to investigate the roles of these factors in the pathogenesis and progression of NS. We further investigated the value of CSF cytokines in diagnosing neurosyphilis.

## 2. Materials and Methods

### 2.1. Patients

This study enrolled 45 patients diagnosed with NS, including 18 patients with ANS and 27 patients with SNS, from May 2018 to June 2019, at the Department of Neurology, Beijing Ditan Hospital, Capital Medical University, and the Department of Neurology, Beijing Tiantan Hospital, Capital Medical University. Twelve patients with syphilis but NNS were also included as controls. All of the enrolled patients were HIV negative. This study was approved by the local ethics committee of Beijing Ditan Hospital, Capital Medical University, and written informed consent was obtained from all participants.

The diagnostic criteria for neurosyphilis was based on the guidelines of CDC in the USA and Europe and related literatures [11–14]. Confirmed neurosyphilis was defined as any stage of syphilis and a reactive RPR in CSF. Presumptive neurosyphilis was defined as syphilis of any stage with (i) a negative CSF RPR test and (ii) reactive CSF *T. pallidum* particle agglutination (TPPA)/fluorescent treponemal antibody absorption (FTA-ABS), and (iii) elevated CSF WBC count (>5/*μ*L) or elevated CSF protein concentration (>45 mg/dL) in the absence of other known causes of these abnormalities. The exclusion criteria were as follows: treatment with antibiotics within the last 1 month, and other infectious diseases (e.g., HIV), autoimmune diseases, and neurodegenerative diseases.

All the enrolled patients underwent lumbar puncture to rule out neurosyphilis, because they either had neurological and/or ophthalmic symptoms/signs or had a serofast status without clinical symptoms/signs. CSF samples were sent for routine examination (i.e., WBC, protein, and glucose concentration) and syphilis testing (i.e., RPR, TPPA, and FTA-ABS). For the purpose of this study, we divided the NS patients into the ANS group and the SNS group according to whether they had clinical symptoms or not.

### 2.2. Biomarker Measurement

The CSF samples were centrifuged and stored at -80°C until biomarker measurements were executed. The samples were simultaneously assayed using the MILLIPLEX® MAP Human High-Sensitivity Cytokine/Chemokine Panels (HSTCMAG-28SK-07; Merck KGaA, Darmstadt, Germany) for the following cytokines: IL-1*β*, IL-4, IL-6, IL-10, IL-17A, IL-21, and TNF-*α*. The minimum detection limits were as follows: IL-1*β*—0.15 pg/mL; IL-4—0.51 pg/mL; IL-6—0.047 pg/mL; IL-10—0.31 pg/mL; IL-17A—0.62 pg/mL; IL-21—0.1 pg/mL; and TNF-*α*—0.39 pg/mL. In addition, the levels of the sTREM2 (ab224881, Abcam) and NFL (CSB-E16094h, CUSABIO) in CSF were determined by ELISA with a minimum detection limit of 10.5 pg/mL and 7.8 pg/mL, respectively.

### 2.3. Statistical Analysis

Statistical analysis was performed by SPSS software (IBM SPSS 25.0 version) or Prism (GraphPad software 7.0 version). Between-group comparisons of continuous data were performed with the nonparametric Mann–Whitney *U* test when the data were nonparametric or independent two-sample *t*-test when the data were parametric. Gender differences were assessed by the chi-square test. Correlations were assessed via Spearman's rank correlation coefficient. Receiver operating characteristic (ROC) curves were used to evaluate the diagnostic values. The Youden index was calculated to determine the cut-off value. A two-sided *p* value < 0.05 was considered statistically significant. The RPR titer measured by the double-dilution method was applied to analyze after a log transformation (log_2_ 1/RPR titer).

## 3. Results

### 3.1. Characteristics of the Patients and CSF Abnormalities

Forty-five patients with NS and 12 patients with NNS were included in this study. The clinical characteristics are summarized in Table 1. Twenty-seven NS individuals had a positive CSF RPR test result (confirmed neurosyphilis). Twenty-one of them had CSF pleocytosis (≥5 cells/*μ*L), and 12 of them had an increased protein CSF concentration (≥45 mg/dL). Eighteen NS individuals had a negative CSF RPR test result (presumptive NS). Fifteen patients from the presumptive NS group had CSF pleocytosis, and 13 patients had elevated CSF protein concentrations. Twenty-seven patients with SNS were enrolled, including those with general paresis (*n* = 18), meningovascular NS (*n* = 5), tabes dorsalis (*n* = 3), and meningeal NS (*n* = 1). Eighteen patients with CSF abnormalities had no neurological symptoms, while they were diagnosed as having ANS.

There was no significant difference in age and gender between the NS group and the NNS group (*p* = 0.112 and *p* = 0.086, respectively). NS patients when compared with NNS patients were characterized by significantly higher levels of serum RPR titer, CSF WBC count, and CSF protein concentration (*p* < 0.001, *p* < 0.001, and *p* < 0.001, respectively). SNS patients had a higher rate of positive CSF RPR than ANS patients (*p* < 0.001). No significant differences were observed regarding the serum RPR titer, CSF WBC count, and CSF protein concentration between SNS and ANS patients.

### 3.2. CSF Cytokine Concentrations

**Figure 1:**
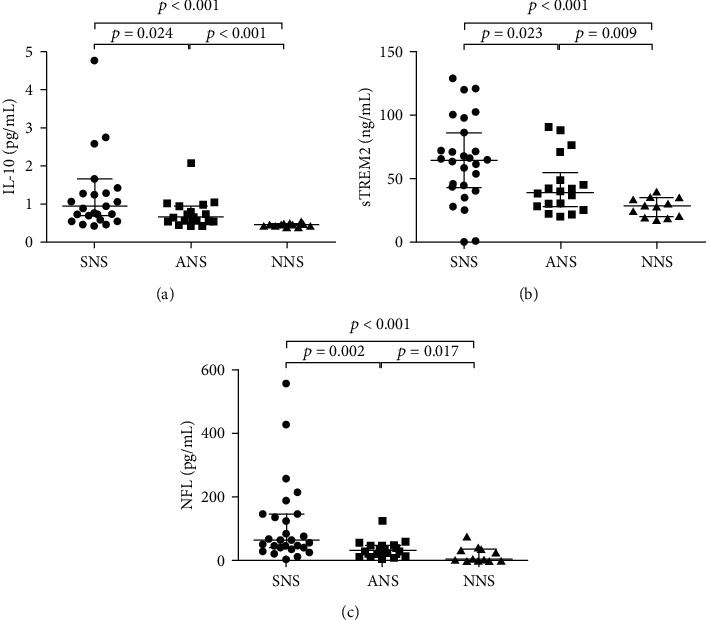
CSF levels of IL-10, NFL, and sTREM2 in ANS, SNS, and NNS. The CSF IL-10 levels were significantly higher in SNS patients than in patients with ANS and NNS, and the CSF IL-10 levels were significantly higher in ANS patients than NNS patients (a). Like CSF IL-10, similar differences between groups were also found in CSF sTREM2 (b) and CSF NFL (c). ANS: asymptomatic neurosyphilis; SNS: symptomatic neurosyphilis; NNS: nonneurosyphilis; IL: interleukin; sTREM2: soluble triggering receptor expressed on myeloid cells 2; NFL: neurofilament light protein.

Patients with NS had significantly higher levels of CSF IL-10 when compared with the NNS group (*p* < 0.001). Notably, in the NS subgroup analysis, the CSF levels of IL-10 were significantly elevated in SNS patients compared to ANS and NNS patients (*p* = 0.024 and *p* < 0.001, respectively), and ANS patients had significantly higher IL-10 levels than NNS patients (*p* < 0.001) (Figure 1(a)). Compared with NNS group, the CSF levels of IL-6 were elevated but did not reach a significant difference (*p* = 0.103). There was a trend of statistical difference in the CSF levels of IL-6 between the SNS group and the NNS group (*p* = 0.055). The CSF levels of IL-17A were below the detection limit. The CSF contents of IL-1*β*, IL-4, IL-21, and TNF-*α* were comparable between the NS group and the NNS group (*p* = 0.307, *p* = 0.220, *p* = 0.651 and, *p* = 0.984, respectively). The CSF levels of cytokines in patients with SNS, ANS, and NNS are shown in Table 2.

### 3.3. CSF Levels of sTREM2 and NFL

The levels of sTREM2 in the CSF of NS patients were significantly higher than those of NNS patients (*p* < 0.001). In the NS subgroup analysis, the CSF levels of sTREM2 were much higher in the SNS group than in the ANS and NNS groups (*p* = 0.023 and *p* < 0.001, respectively). The CSF levels of sTREM2 were also significantly higher in the ANS group than in the NNS group (*p* = 0.009) (Figure 1(b)).

The NFL levels in the CSF of NS patients were also significantly increased compared with those of NNS patients (*p* < 0.001). When we analyzed the NS patients separately, the CSF NFL levels in the SNS group were significantly higher than those in the ANS and NNS groups (*p* = 0.002 and *p* < 0.001, respectively). The CSF NFL levels in ANS patients were also higher than those in NNS patients (*p* = 0.017) (Figure 1(c)). The CSF levels of sTREM2 and NFL in patients with SNS, ANS, and NNS are shown in Table 2.

### 3.4. Correlations of CSF IL-10 with CSF sTREM2 and NFL

**Figure 2:**
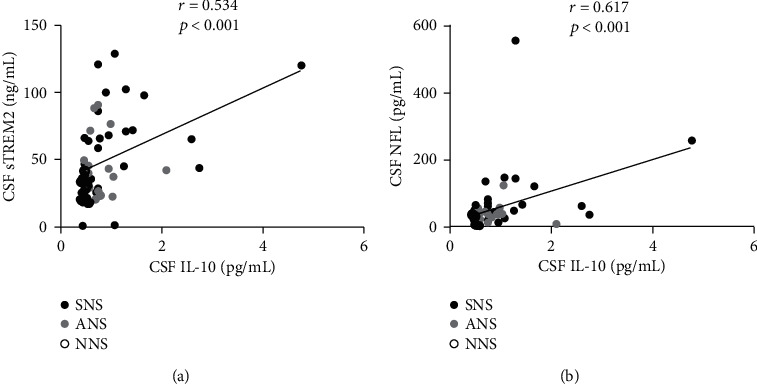
Positive correlations of CSF IL-10 with CSF sTREM2 (a) and CSF NFL (b). *r*: Spearman's rho. CSF: cerebrospinal fluid; IL: interleukin; NFL: neurofilament light protein; sTREM2: soluble triggering receptor expressed on myeloid cells 2; ANS: asymptomatic neurosyphilis; SNS: symptomatic neurosyphilis; NNS: nonneurosyphilis.

We explored the correlations of the levels of CSF IL-10 with CSF sTREM2 and CSF NFL in all patients using the Spearman rank correlation test. Positive correlations were found as follows: CSF IL-10 and CSF sTREM2 (*r* = 0.534, *p* < 0.001) (Figure 2(a)) and CSF IL-10 and CSF NFL (*r* = 0.617, *p* < 0.001) (Figure 2(b)).

### 3.5. Correlations of CSF IL-10 with CSF Laboratory Data and Serum RPR

**Figure 3:**
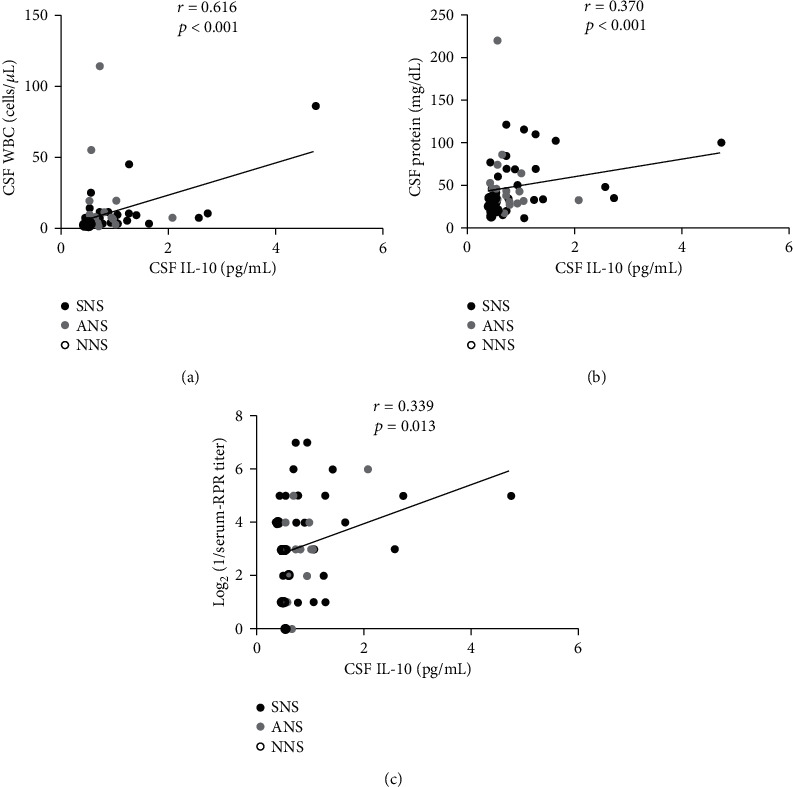
Positive correlations of CSF IL-10 with CSF WBC (a), CSF protein (b) and log_2_ (1/serum RPR titer). *r*: Spearman's rho. CSF: cerebrospinal fluid; IL: interleukin; WBC: white blood cell; RPR: rapid plasma reagin; ANS: asymptomatic neurosyphilis; SNS: symptomatic neurosyphilis; NNS: nonneurosyphilis.

CSF levels of IL-10 were positively correlated with CSF WBC count (*r* = 0.616, *p* < 0.001) (Figure 3(a)) and CSF protein concentration (*r* = 0.370, *p* < 0.001) (Figure 3(b)). We also found a positive correlation between CSF IL-10 and log_2_ (1/serum RPR titer) (*r* = 0.339, *p* = 0.013) (Figure 3(c)).

### 3.6. Diagnostic Value of CSF IL-10 Levels for NS

**Figure 4:**
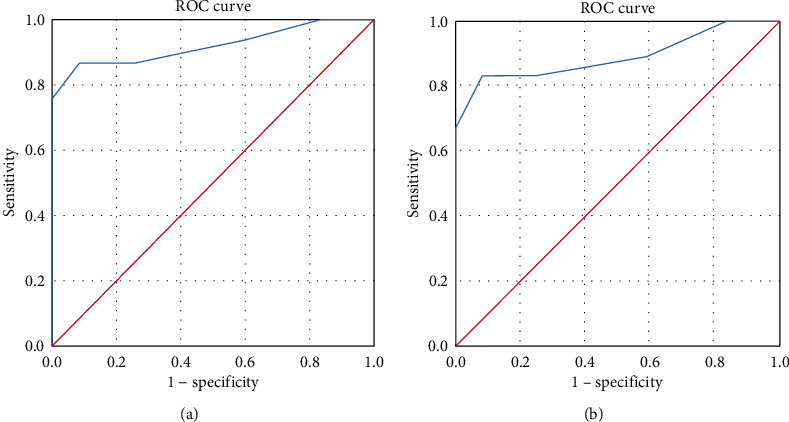
ROC curves of CSF IL-10. (a) ROC curve of CSF IL-10 in the diagnosis of neurosyphilis (NS); AUC = 0.920 (95% CI 0.852-0.989, *p* < 0.001). (b) ROC curve of CSF IL-10 in the diagnosis of ANS; AUC = 0.891 (95% CI 0.772-1.000, *p* < 0.001). ROC: receiver operator characteristics; AUC: areas under the ROC curve; CI: confidence interval; IL: interleukin.

Given the marked elevation of CSF IL-10 levels in NS patients, we further assessed the diagnostic value of CSF IL-10 as a marker in NS using ROC curve analysis. The results showed that the area under the ROC curve (AUC) was 0.920 (95% CI 0.852-0.989, *p* < 0.001) (Figure 4(a)). The optimal cut-off point was defined by the sum of maximum sensitivity and specificity, which was 0.53 pg/mL. The sensitivity and specificity of this cut-off were 86.7% and 91.7%, respectively.

We also estimated the diagnostic values of CSF IL-10 in ANS. The results showed that the AUC was 0.891 (95% CI 0.772-1.000, *p* < 0.001) (Figure 4(b)), and the optimal cut-off point was 0.53 pg/mL with a sensitivity of 83.3% and a specificity of 91.7%.

### 3.7. Diagnostic Value of CSF sTREM2 and NFL Levels for NS

The CSF sTREM2 and NFL levels significantly discriminated NS from NNS (AUC = 0.831, 95% CI 0.728-0.935, *p* < 0.001; AUC = 0.826, 95% CI 0.684-0.968, *p* = 0.001; respectively) (Supplementary Figures 1a, b). The optimal cut-off points for sTREM2 and NFL were 40.64 ng/mL and 8.29 pg/mL, respectively. The sensitivity/specificity of sTREM2 and NFL were 66.7%/100% and 95.6%/58.3%, respectively. The CSF sTREM2 and NFL levels could also significantly discriminate ANS from NNS (AUC = 0.764, 95% CI 0.595-0.933, *p* = 0.016; AUC = 0.745, 95% CI 0.548-0.943, *p* = 0.025; respectively) (Supplementary Figures 1c, d). The optimal cut-off point for sTREM2 was 36.54 ng/mL with a sensitivity of 61.1% and a specificity of 91.7%. The optimal cut-off point for NFL was 8.29 pg/mL with a sensitivity of 94.4% and a specificity of 58.3%.

## 4. Discussion

*T. pallidum* invades the CNS within days after primary infection [5], and those who fail to remove *T. pallidum* by itself were ANS, and may progress into SNS [6, 7]. In some patients, even if sufficient penicillin was given in the early stage of syphilis, it did not prevent the occurrence of NS. At present, the pathogenesis of neurosyphilis is largely unknown, and immune response may participate in the whole process of neurosyphilis. Recent studies have shown that IL-17A and IFN-*γ* were involved in the proinflammatory immune response in NS [15]. IL-10 is a potent anti-inflammatory cytokine, and excessive production of IL-10 has been observed in late syphilis [16]. The evidence also showed that CSF IL-10 levels were elevated in NS [10]. Very few studies have been published on the comparison of the immune response in ANS and SNS patients.

In this study, we explored the levels of CSF proinflammatory and anti-inflammatory cytokines in patients with NS, and the correlations of these cytokines with markers of microglial activation and neuronal injury. In addition, we investigated differences of these biomarkers in ANS and SNS patients, and the diagnostic value of these cytokines was also explored.

Of the measured cytokines in the CSF, only IL-10 was significantly elevated in NS patients compared to NNS patients, which is identical with the results of other studies [10]. IL-10 is an anti-inflammatory cytokine. During infection, not only does it prevent inflammatory processes, which play an important role in the pathogen clearance, as discussed elsewhere [17–19], but it also contributes to immunopathologic lesions with pathogens [20–23]. Nevertheless, excessive production of IL-10, as observed in infections caused by a number of pathogens, such as HIV [24, 25], hepatitis C virus [26], and mycobacteria [27], can inhibit proinflammatory response that enables pathogens to escape immune control, resulting in either fatal or chronic nonhealing infections [28]. Whether excessive production of IL-10 during infections is a cause or a consequence of high pathogen burdens is not often clear. In either case, high expression of IL-10 can enable *T. pallidum* to escape immune clearance and may cause persistent infection of *T. pallidum*. We found that SNS patients had significantly higher CSF IL-10 levels than ANS patients, suggesting that IL-10 production was associated with neurological symptoms and progression of disease. The levels of CSF IL-10 were correlated with CSF WBC count, CSF protein concentration, and CSF RPR titer, indicating that CSF IL-10 may be involved in the inflammation process in NS patients. Furthermore, we found that the CSF IL-10 levels had positive correlation with the neuronal injury marker NFL, confirming that high concentrations of IL-10 play an important role in the progression of the disease.

Microglia are the “inspectors” of CNS, which continuously monitor the CNS environment, responding to any disturbance in neuronal homeostasis. Once harmful stimuli, such as infection, trauma, and toxic stimuli, are identified, microglia are activated rapidly [29–31]. At different stages of the development of neurological diseases, microglia may have a spectrum of phenotypes, which may be related to the change of the surrounding microenvironment. The two extremes of the activated microglia are M1 and M2 polarized cells [32]. The M1 phenotype is characterized by the production of proinflammatory cytokines (IL-6, IL-1*β*, and TNF-*α*) [33, 34], while the M2 phenotype is characterized by the production of anti-inflammatory cytokines (IL-10 and IL-4) [35, 36]. Microglia activation is an important pathological feature of NS [37, 38]. Our study found that the levels of microglia activation marker sTREM2 in the CSF of SNS patients were higher than those of ANS patients. This indicated a more obvious microglia activation in patients with SNS. We found that the CSF levels of IL-10 in NS patients were correlated to the CSF levels of sTREM2, suggesting that CSF IL-10 was mainly produced by activated microglia.

IL-10 has become a potential therapeutic target for several neurological disorders. The intracranial administration of IL-10 improved the outcome of EAE [39]. However, several studies showed the detrimental effects of IL-10 in Alzheimer's disease. For example, the administration of IL-10 worsened the outcome of the amyloid precursor protein transgenic mice by reduced amyloid-*β* phagocytosis by microglia [40], while the absence of IL-10 reduced the amyloid-*β* load in the transgenic mouse brains, via phagocytosis by activated microglia [41]. Therefore, inhibitory actions or production of the key anti-inflammatory cytokine IL-10 may be a potential treatment strategy for neurosyphilis patients with abnormal immunity.

At present, the diagnosis for NS remains a major clinical challenge. There is no “gold standard” to establish or exclude the diagnosis of NS. The laboratory diagnosis of NS is based on the abnormal results of CSF immunologic tests and on the elevation of CSF WBC count and protein concentrations. A reactive CSF Venereal Disease Research Laboratory (VDRL) test is generally considered specific for NS. However, the CSF VDRL test has low sensitivity [5, 42]. In this study, we used RPR instead of VDRL. Several studies and European guidelines have shown that the RPR test is an alternative to VDRL [14, 43, 44]. The diagnosis of NS with neurological symptoms and signs (i.e., SNS) is not difficult. However, the diagnosis of ANS, especially with nonreactive CSF RPR, can be difficult and uncertain. As the laboratory diagnosis of NS is difficult, new markers are needed. Our study indicated that CSF levels of IL-10, sTREM2, and NFL might present useful diagnostic value for NS and ANS. The diagnostic accuracy of CSF IL-10 was slightly higher than that of CSF sTREM2 and NFL, with both high sensitivity and specificity.

Some limitations in our study need to be mentioned. First, the sample size was relatively small. The studies should be verified in a large-scale patient population. Second, the results of blood proinflammatory and anti-inflammatory cytokine profiles were lacking because of the absence of serum samples. Third, the dynamic changes of these cytokines after treatment should be included in this study. Further studies are expected to address these issues.

## 5. Conclusions

The study found that the CSF IL-10 levels in NS patients were significantly increased compared to those in NNS patients, and they peaked in SNS patients. The CSF IL-10 levels had positive correlation with microglial activation and neuronal injury markers and also with CSF RPR titer, CSF WBC count, and CSF protein concentration. These results indicate that the excessive production of CSF IL-10 might facilitate bacterial persistent infection and play an important role in the pathogenesis of NS; it might also be associated with the progression of the disease. In addition, we also found that CSF IL-10 concentration had a useful value in the diagnosis of NS, especially in ANS.

## Figures and Tables

**Table 1:** Clinical characteristics of patients with ANS, SNS, and NNS.

Clinical characteristics	NS (*n* = 45)		NNS (*n* = 12)
	SNS (*n* = 27)	ANS (*n* = 18)	
Sex ratio (*n*) (M/F)	20/7	12/6	5/7
Age (years)	52.04 ± 12.99	45.33 ± 10.73	42.67 ± 13.79
Serum RPR titer	1 : 16 (1 : 4-1 : 32)	1:8 (1 : 8-1 : 16)	1 : 2 (1 : 1-1 : 8)
Positive CSF RPR (*n*)	22/27 (81.5%)^a^	5/18 (27.8%)	0/12 (0%)
Positive CSF TPPA (*n*)	27/27 (100%)	18/18 (100%)	0/12 (0%)
Positive CSF FTA-ABS (*n*)	27/27 (100%)	18/18 (100%)	0/12 (0%)
CSF WBC count (/*μ*L)	8.00 (5.00-15.00)	6.50 (3.75-14.00)	2.50 (1.25-3.00)
CSF protein level (mg/dL)	48.30 (33.70-84.50)	40.25 (31.28-55.90)	22.25 (17.90-32.42)

Values are expressed as median (IQR), unless otherwise indicated. NS: neurosyphilis; ANS: asymptomatic neurosyphilis; SNS: symptomatic neurosyphilis; NNS: nonneurosyphilis; M: male; F: female; RPR: rapid plasma reagin; CSF: cerebrospinal fluid; TPPA: pallidum particle agglutination; FTA-ABS: fluorescent treponemal antibody absorption. WBC: : white blood cell. ^a^*p* < 0.05 compared with ANS.

**Table 2:** CSF biomarker levels of patients with ANS, SNS, and NNS.

	NS (*n* = 45)		NNS (*n* = 12)
	SNS (*n* = 27)	ANS (*n* = 18)	
IL-1*β* (pg/mL)	0.10 (0.09-0.12)	0.10 (0.09-0.12)	0.11 (0.10-0.12)
IL-4 (pg/mL)	1.05 (0.90-1.89)	1.05 (0.90-1.42)	1.29 (0.94-2.07)
IL-6 (pg/mL)	1.16 (0.49-2.21)	0.75 (0.41-2.09)	0.52 (0.45-1.01)
IL-10 (pg/mL)	0.95 (0.70-1.66)^a,b^	0.68 (0.55-0.96)^c^	0.47 (0.44-0.50)
IL-21 (pg/mL)	0.42 (0.35-0.43)	0.38 (0.31-0.46)	0.41 (0.37-0.47)
TNF-*α* (pg/mL)	0.54 (0.47-0.75)	0.54 (0.43-0.70)	0.61 (0.51-0.63)
sTREM2 (ng/mL)	65.01 (43.59-86.25)^a,b^	39.63 (28.00-55.07)^c^	28.75 (20.19-35.33)
NFL (pg/mL)	64.08 (40.01-146.82)^a,b^	33.79 (13.92-49.44)^c^	5.71 (0.18-35.23)

Values are expressed as median (IQR). NS: neurosyphilis; ANS: asymptomatic neurosyphilis; SNS: symptomatic neurosyphilis; NNS: nonneurosyphilis; IL: interleukin; TNF: tumor necrosis factor; sTREM2: soluble triggering receptor expressed on myeloid cells 2; NFL: neurofilament light protein. ^a^*p* < 0.05 compared with ANS. ^b^*p* < 0.05 compared with NNS. ^c^*p* < 0.05 compared with NNS.

## Data Availability

The data used to support the findings of this study are available from the corresponding authors upon request.

## Supplementary Materials

Supplementary Materials Supplementary Figure 1: ROC curves of CSF sTREM2 and NFL.
