# Supplementary material for: Cerebrospinal Fluid Cytokines in Patients with Neurosyphilis: The Significance of Interleukin-10 for the Disease
Source: Biomed Res Int. 2020 Oct 1;2020:3812671. doi: 10.1155/2020/3812671 (PMC7556108; doi:10.1155/2020/3812671)
Supplement: Supplementary Materials — Supplementary Figure 1: ROC curves of CSF sTREM2 and NFL. [file 3812671.f1.docx]

Supplementary Figure 1. ROC curves of CSF sTREM2 and NFL.


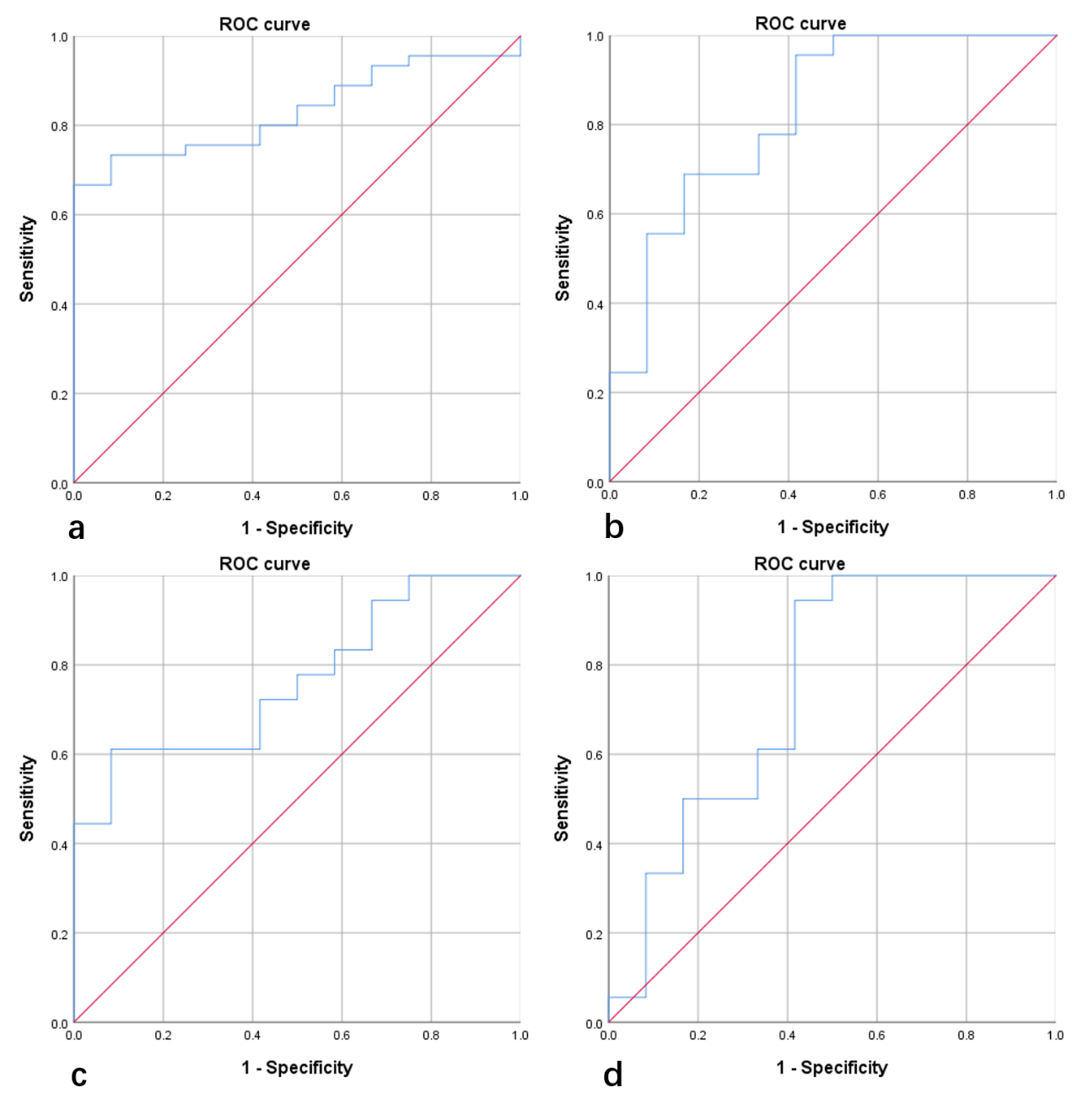


**a** ROC curves of CSF sTREM2 in the diagnosis of NS, AUC=0.831 (95%CI 0.728-0.935, p<0.001). **b** ROC curves of CSF NFL in the diagnosis of NS, AUC=0.826 (95%CI 0.684-0.968, p=0.001). **c** ROC curves of CSF sTREM2 in the diagnosis of asymptomatic neurosyphilis (ANS), AUC=0.764 (95%CI 0.595-0.933, p=0.016). **d** ROC curves of CSF NFL in the diagnosis of ANS, AUC=0.745 (95%CI 0.548-0.943, p=0.025). ROC=Receiver operator characteristics; AUC= areas under the ROC curve; CI=confidence interval; sTREM2, soluble Triggering receptor expressed on myeloid cells 2; NFL, neurofilament light protein.
